# A Study on Neonatal Intake of Oleanolic Acid and Metformin in Rats (*Rattus norvegicus*) with Metabolic Dysfunction: Implications on Lipid Metabolism and Glucose Transport

**DOI:** 10.3390/molecules23102528

**Published:** 2018-10-03

**Authors:** Mmahiine Molepo, Ademola Ayeleso, Trevor Nyakudya, Kennedy Erlwanger, Emmanuel Mukwevho

**Affiliations:** 1 Department of Biochemistry, Faculty of Natural and Agricultural Science, North West University, Mafikeng Campus, Private Bag X2046, Mmabatho 2735, South Africa; chrissy.molepo@gmail.com; 2 Department of Biochemistry, Faculty of Science, Adeleke University, Ede 232, P.M.B. 250, Osun State, Nigeria; ademola.ayeleso@adelekeuniversity.edu.ng; 3 Department of Human Anatomy and Physiology, Faculty of Health Sciences, University of Johannesburg, Doornfontein, Johannesburg 2028, South Africa; trevorn@uj.ac.za; 4 School of Physiology, Faculty of Health Sciences, University of the Witwatersrand, Parktown, Johannesburg 2193, South Africa; Kennedy.Erlwanger@wits.ac.za

**Keywords:** metabolic syndrome, oleanolic acid, metformin, lipid metabolism, glucose transport

## Abstract

Metabolic syndrome, a cluster of different disorders which include diabetes, obesity and cardiovascular diseases, is a global epidemic that is growing at an alarming rate. The origins of disease can be traced back to early developmental stages of life. This has increased mortalities and continues to reduce life expectancies of individuals across the globe. The aim of this study was to investigate the sub-acute and long term effects of neonatal oral administration of oleanolic acid and metformin on lipids (free fatty acids, FFAs) and genes associated with lipid metabolism and glucose transport using a neonatal rat experimental model. In the first study, seven days old pups were randomly grouped into control—distilled water (DW); oleanolic acid (60 mg/kg), metformin (500 mg/kg), high fructose diet (20% *w*/*v*, HF), oleanolic acid (OA) + high fructose diet (OA + HF), and Metformin + high fructose diet (MET + HF) groups. The pups were treated for 7 days, and then terminated on postnatal day (PD) 14. In the second study, rat pups were initially treated similarly to study 1 and weaned onto normal rat chow and plain drinking water on PD 21 till they reached adulthood (PD112). Tissue and blood samples were collected for further analyses. Measurement of the levels of free fatty acids (FFAs) was done using gas chromatography-mass spectrometry. Quantitative polymerase chain reaction (qPCR) was used to analyze the gene expression of *glut-4*, *glut-5*, *fas*, *acc-1*, *nrf-1* and *cpt-1* in the skeletal muscle. The results showed that HF accelerated accumulation of saturated FFAs within skeletal muscles. The HF fed neonatal rats had increased stearic acid, which was associated with decreased glucose, suppressed expression of *glut-4*, *glut-5*, *nrf-1* and *cpt-1* genes, and increased expression of *acc-1* (*p* < 0.01) and *fas*. OA + HF and MET + HF treated groups had increased mono- and polyunsaturated FFAs; oleic, and octadecadienoic acids than the HF group. These unsaturated FFAs were associated with increased *glut-4*, *glut-5* and *nrf-1* (*p* < 0.01) and decreased *acc-1* and *fas* (*p* < 0.05) in both OA + HF and MET + HF treated groups. Conclusions: The present study shows that neonatal oral administration of oleanolic acid and metformin potentially protects against the development of fructose-induced metabolic dysfunction in the rats in both short and long time periods.

## 1. Introduction

The World Health Organization (WHO) has indicated that the world’s population living with obesity has doubled to 13% since the 1980s, affecting more than 600 million adults and 42 million children under the age of 5 years in 2014 [1]. This indicates that obesity has become a global epidemic that is growing at an alarming rate. As a risk factor to metabolic syndrome, obesity results in high mortalities both in children and adults, and continues to reduce life expectancies of individuals across the globe [2]. Metabolic syndrome occurs when there is a disruption of metabolic functions in which essential substances such as hormones, enzymes, proteins, or genes are expressed or produced in low or high concentrations in the body causing the metabolic network to fail [3,4]. This means that metabolic syndrome occurs when there are abnormalities in the metabolic system. Initially, interventions in lifestyle including adopting a healthy diet, increasing physical activity and weight loss were recommended as the most effective mitigation of obesity and metabolic syndrome [5]. However, in some cases, even when individuals manage to lose weight, they still continue to suffer from metabolic syndrome. Obesity is the sixth most lethal risk factor contributing to a number of metabolic diseases [1,6]. Obesity is a complicated condition which is not easy to mitigate, as it is not only caused by excess food intake and comparatively less energy expenditure, but complex metabolic disruptions that are centered on lipid metabolism and cellular signaling systems linked to it [7].

Pharmacotherapy has been recommended as an effective way to counter metabolic syndrome associated with obesity [5]. However, it has been reported that most pharmacological anti-obesity drugs which also tend to be expensive, have side effects such as altering blood pressure, headaches, dizziness and other unbearable collateral effects that adversely affect the quality of life [2]. There is a growing need for less costly, effective anti-obesity agents that have minimal collateral effects. Phytochemicals are increasingly being explored for their therapeutic potential. An example of such is oleanolic acid (3β-hydroxy-olean-12-en-28-oic acid), which is a natural, bioactive phytochemical component found in several plant foods and medicinal herbs [8,9]. It is a pentacyclic triterpenoid that is found in abundance in plants of the Oleaceae family such as the olive plant and also occurs in nature as a free acid or as an aglycone of triterpenoid saponins [10]. It is biosynthesized by the acetate/mevalonate pathway and (3S)-2,3-oxidosqualene cyclization [8].

Metformin is a widely used pharmaceutical drug for treating components of metabolic syndrome, and was discovered five decades ago [11]. It is an orally administered anti-diabetic drug from the biguanide class, which has been proven to reduce plasma insulin, glucose, FFAs and overall body weight in diabetic and obese patients [12]. Oleanolic acid and metformin exhibit several pharmacological properties such as antioxidant, microbicide, antidiabetic, anti-inflammatory, hypolipidaemic antiatherosclerotic and anti-cancer actions [8,9,11,13]. The neonatal period is a phase of life with developmental plasticity wherein interventions have long term impacts on the health and disease outcomes of individuals. Hence, the aim of this study was to examine the sub-acute and long term potential protective effects of neonatal oral administration of oleanolic acid and metformin against fructose-induced alterations in the level of lipids (free fatty acids, FFAs) and impaired expression of genes associated with lipid metabolism and glucose transport using a rat model.

## 2. Results

### 2.1. Growth Development of the Rats

In the first study, there was a significant increase in body mass from the induction of treatments (PD7) up to termination across all treatment groups (*p* < 0.05; Table 1). There were no significant differences across all treatment groups at induction and termination (*p* > 0.001).

**Figure 1:**
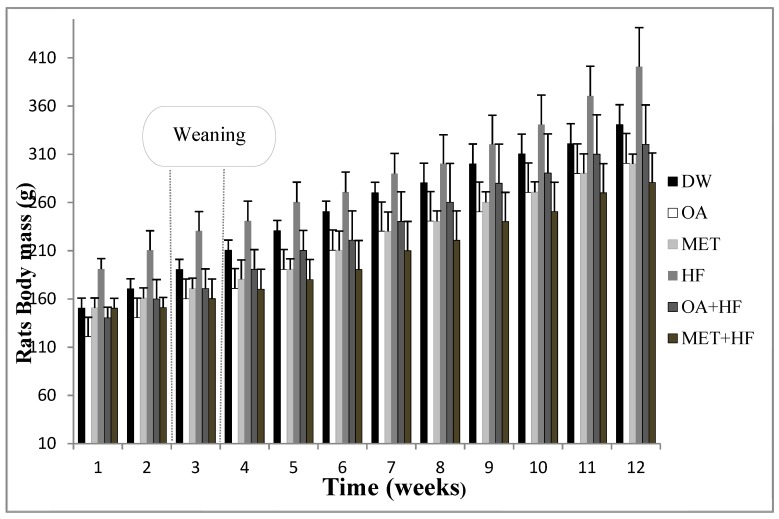
Weekly body mass measurements from induction of treatments to termination in the long term study 2. Rats weight development of Phase 2 experiment. From Phase 1 to Phase 2 of the experiment, there was a statistically significant difference in weight development (n = 8) (DW *p* < 0.001, OA *p* < 0.001, MET *p* < 0.001, HF *p* < 0.001, OA + HF *p* < 0.001 and MET + HF *p* < 0.001). Error bars indicate the 95% confidence intervals based on the rats weight’s student t distribution.

**Figure 2:**
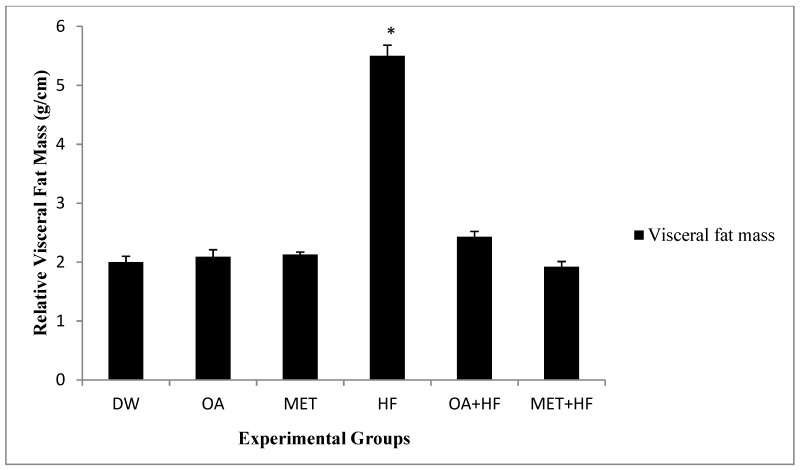
The effect of neonatal oral administration of oleanolic acid and metformin on visceral fat mass in female rats fed a high fructose diet. All experimental groups were compared with the control (DW); HF *p* < 0.05 (*) (n = 8).

In the second study, the rats grew significantly from induction at PD7, to weaning PD21 and subsequently to termination age PD112 (*p* < 0.05; Figure 1). The rats that received fructose in the neonatal phase and adulthood had significantly higher terminal body masses compared to the other treatment groups (*p* < 0.05; Figure 1). Oleanolic acid and MET treatment in the neonatal period (OA, MET, OA + HF and MET + HF) attenuated the fructose induced increase in terminal body mass (*p* < 0.05; Figure 1). Moreover, there was no distinct significant difference in visceral fat mass between the control and OA, MET, OA + HF and MET + HF (Figure 2). However, there was a significant difference between the control and the HF group (*p* < 0.05; Figure 2).

### 2.2. Saturated Free Fatty Acid

In the sub-acute study (Experiment 1), the levels of stearic acid were significantly higher in the group that was treated with fructose (HF) compared to the other treatment groups (*p* < 0.05; Table 2). Neonatal OA and MET treatment prevented the fructose induced increase in stearic acid (*p* < 0.05; Table 2). In the long term study (Experiment 2), stearic acid levels were 5-fold higher in the rats that received HF compared to treatment groups (*p* < 0.05; Table 2). Neonatal oral administration of OA and MET prevented the fructose induced increase in stearic acid (*p* < 0.01; Table 2).

The stearic acid response factor was lower in all long term (study 2) experimental groups than that of the sub-acute study (Table 2). However, only the control groups and the HF groups were statistically significant (DW *p* < 0.05 and HF *p* < 0.01). Moreover, there was no statistical significant difference in the overall response factor of acid from both experiments 1 and 2. Stearic acid was higher in the HF group, which is the group where obesity/metabolic dysfunctions were induced. These suggest that HF promotes accumulation of saturated free fatty acids in the body.

### 2.3. Monounsaturated Free Fatty Acids

In Study 1, oleic acid was significantly higher in the OA (1.55 ± 1.03; *p* < 0.001) and OA + HF group (0.09 ± 0.08; *p* < 0.05) compared to the other treatment groups. Oleic acid was not detected from the control group, MET, HF and MET + HF.

In Study 2, oleic acid was not detected in the control group and the HF group (Table 3). However, it was detected in the OA (1.39 ± 0.26), MET (0.67 ± 0.47), OA + HF (0.17 ± 0.03) and the MET + HF (0.06 ± 0.03) groups. The OA, MET and OA + HF groups were significantly different from the control group (OA *p* < 0.001, MET *p* < 0.001 and OA + HF *p* < 0.05). Moreover, OA (1.39 ± 0.26) and MET (0.67 ± 0.47) groups had high data dispersions than the OA + HF group (0.17 ± 0.03) (Table 3). Oleic acid was higher in both the OA and the OA + HF groups (Table 3). The MET group was the only group that was significantly different between experiment 1 and 2 (*p* < 0.001). Oleic acid was not detected in the sub-acute study rats of the MET group and MET + HF group, but it was detected in the groups of the long term experiment rats. The response factor of oleic acid from all samples of both sub-acute and long term experiments was statistically significantly different (*p* < 0.01). Both in study 1 and 2 of the experiment, oleic acid was prominent in the OA and OA + HF groups.

### 2.4. Polyunsaturated Free Fatty Acid

In Study 1, octadecadienoic acid was highly responsive in the OA group (*p* < 0.001), resulting in a response factor 7-fold significantly higher than that of the control group (Table 4). The MET group (*p* < 0.001) also had a 2-fold significantly higher response factor as compared with the control group (Table 4). The octadecadienoic acid response factor in the OA group (2.96 ± 3.12) was also 2-fold higher than that of MET group (1.07 ± 0.60). The HF group (*p* < 0.05) had significantly lower levels of octadecadienoic acid as compared with the control and with the rest of the experimental groups (Table 4). The OA + HF group (0.43 ± 0.58) and MET + HF group (0.51 ± 0.53) were not distinctively different from each other and were also not statistically different from the control group (Table 4).

In Study 2, only the OA group was statistically significantly higher than the control group (*p* < 0.001), the MET group (0.75 ± 0.55) was higher than the control group (0.43 ± 0.58) but not significant (Table 4). In the HF group, there was no octadecadienoic acid detected (Table 4). The OA + HF (0.24 ± 0.17) and MET + HF (0.21 ± 0.15) groups were not distinctively different from each other and were both lower than the control group but not significant (Table 4). When the Study 1 octadecadienoic acid response factor was compared with that of Study 2, the experimental groups that were statistically significantly different from each other were the OA, HF, OA + HF and MET + HF groups (OA *p* < 0.01, HF *p* < 0.05, OA + HF *p* < 0.05 and MET + HF *p* < 0.05). The control group response factor of study 2 (0.43 *Ra*) was slightly higher than that of Study 1 (0.35 *Ra*) (Table 4). There was a statistically significant difference in the response factor of octadecadienoic acid from all studies of the experiment (*p* < 0.01). The polyunsaturated free fatty acid was consistently highly responsive in the OA and MET groups. OA + HF and MET + HF groups were higher than that of HF, but not distinctive from one another. The OA diet promoted the accumulation of polyunsaturated free fatty acids in the body more than the MET diet did.

### 2.5. Effects of Neonatal OA and MET Treatment on the Expression of Glut-4

**Figure 3:**
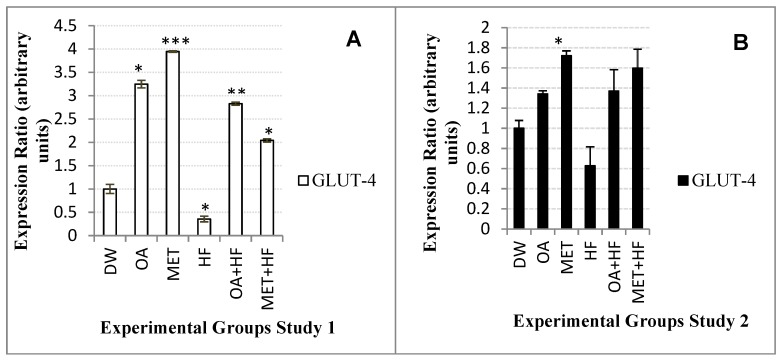
*Glut-4* gene expression in the skeletal muscle of rats in study 1 (**A**) and study 2 (**B**) of the experiment, in which each group was compared with the control (DW) (n = 3); *p* < 0.05 (*), *p* < 0.01 (**) and *p* < 0.001 (***).

In Study 1, the MET group (*p* < 0.001) *glut-4* expression was 4-fold significantly higher than that of the control group and in the OA group (*p* < 0.01), it was 3-fold significantly higher than the control group (Figure 3A). Moreover, the OA + HF (*p* < 0.01) and MET + HF (*p* < 0.05) groups were also higher than the control group by 2.5-fold and 2-fold (Figure 3A).

In Study 2 experiment, *glut-4* expression from the OA, MET (*p* < 0.05), OA + HF and MET + HF groups were higher than the control group, but were all lower than that of Study 1 (Figure 3A,B). *Glut-4* expression in the HF group was lower than that of the control and lower than the rest of the experimental diet groups (Figure 3A,B). The HF group suppressed the expression of *glut-4* gene in both studies 1 and 2 of the experiment. The MET experimental diet increased the expression of *glut-4* better than OA experimental diet in both study 1 and 2 experiments. In both studies, the expression of *glut-4* gene was higher in the MET group and the OA group (Figure 3A,B).

### 2.6. Effects of Neonatal OA and MET Treatment on the Expression of Nrf-1

**Figure 4:**
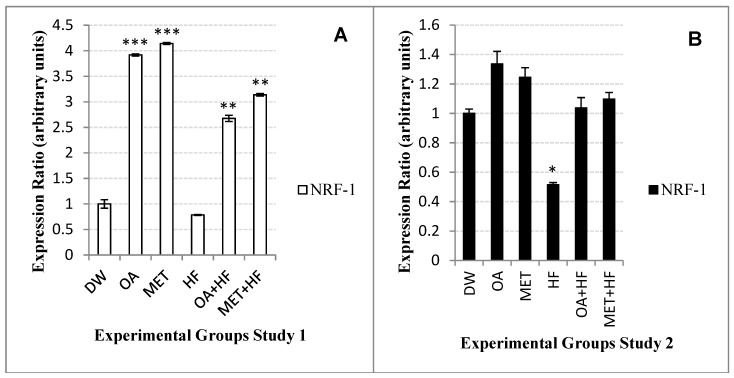
*Nrf-1* gene expression in the skeletal muscle of rats in Study 1 (**A**) and Study 2 (**B**) (n = 3) experimental skeletal muscle samples of the study. Each experimental group was compared with DW (control) in each study; *p* < 0.05 (*), *p* < 0.01 (**) and *p* < 0.001 (***).

There was a 3.9-fold increase in the expression of *nrf-1* in the OA (*p* < 0.001) group and an increase of 4-fold in the MET group (*p* < 0.001), in the Study 1 experiments (Figure 4A). The OA + HF (*p* < 0.01) had an expression of *nrf-1* at about 2.5-fold, whilst MET + HF group (*p* < 0.01) had about 3-fold increase as compared with the control group (Figure 4A).

In the Study 2 experiments, the overall expression of *nrf-1* was lower than 1.5 expression ratio, with the OA, MET, OA + HF and MET + HF groups being higher than that of the control group (Figure 4B). In the HF group (*p* < 0.05), the expression of *nrf-1* was significantly suppressed to 0.78 expression ratio in Study 1 whereas in Study 2 it was suppressed to 0.51 expression ratio (Figure 4A,B). As a result, the HF group decreased the *nrf-1* expression in both studies of the experiment. However, OA and MET both increased the expression of *nrf-1*, with the expression of MET group being the highest in study 1 and OA dietary group being the highest in Study 2 (Figure 4A,B). Moreover, the MET + HF *nrf-1* expression was higher than that of OA + HF in both studies 1 and 2 of the experiment (Figure 4A,B).

### 2.7. Effects of Neonatal OA and MET Treatment on the Expression of Acc-1

**Figure 5:**
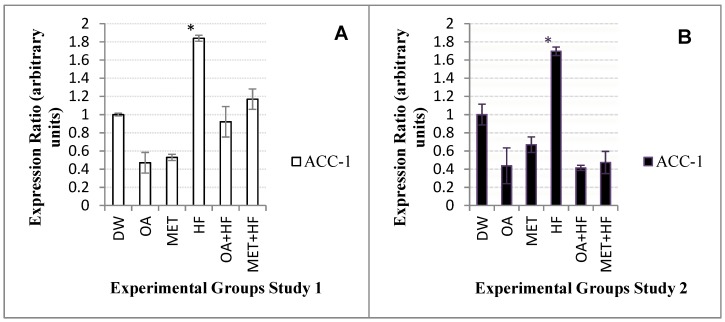
*Acc-1* gene expression in skeletal muscle samples of rats in study 1 (**A**) and study 2 (**B**) of the experiment. Each group versus the control group (DW) (n = 3); *p* < 0.05 (*).

In the sub-acute study of the experiment, *acc-1* expression was decreased in the OA group by about 1-fold, in MET group by 1-fold and in OA + HF group by 0.1-fold from that of the control group (Figure 5A). The MET + HF group was about 0.16 higher than the control (Figure 5A). In both Study 1 and Study 2, the HF group (*p* < 0.05) had a significantly increased expression, of about 1-fold higher compared to the control group (Figure 5A,B).

In Study 2 of the experiment, the expression of *acc-1* from the OA, OA + HF and MET + HF groups were about 1-fold higher than that of the control (Figure 5B). The MET dietary group also decreased the expression of *acc-1* gene (Figure 5B).

### 2.8. Effects of Neonatal OA and MET Treatment on the Expression of Fas

**Figure 6:**
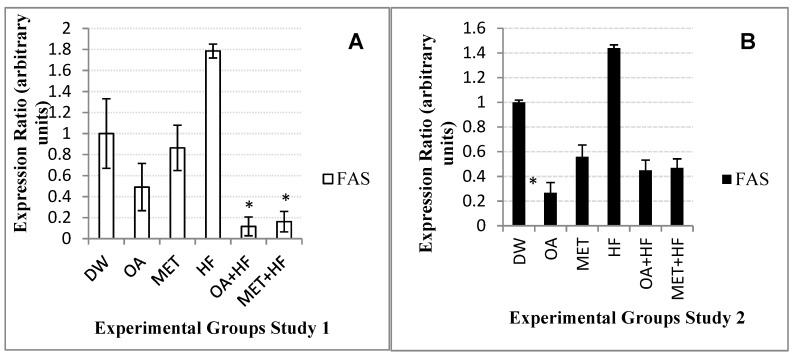
*Fas* gene expression in the skeletal muscle of rats in Study 1 (**A**) and Study 2 (**B**) of the experiment. Each group was compared with DW (control group) (n = 3); *p* < 0.05 (*).

The expression of *fas* gene was about 1-fold lower than the control group in the OA group, whereas in the MET + HF it was 0.9 lower than the control group in Study 1 of the experiment (Figure 6A). The OA + HF (*p* < 0.05) and MET + HF (*p* < 0.05) groups had an expression of *fas* of about 8-fold and 5-fold significantly lower than that of the control group (Figure 6A).

In Study 2 of the experiment, the expression ratio of *fas* in the OA group (*p* < 0.05) was significantly decreased by 3-fold, whereas the expression ratio in both OA + HF and MET + HF groups were just 1-fold less than that of the control group (Figure 6B). However, in Study 1 the expression of *fas* was about 14-fold lower than of the control in the OA + HF group, and that of the MET + HF group was 8-fold lower (Figure 6A). The HF group increased the expression of FAS in both studies 1 and 2 of the experiment (Figure 6A,B).

### 2.9. Effects Neonatal OA and MET Treatment on Expression of Cpt-1

**Figure 7:**
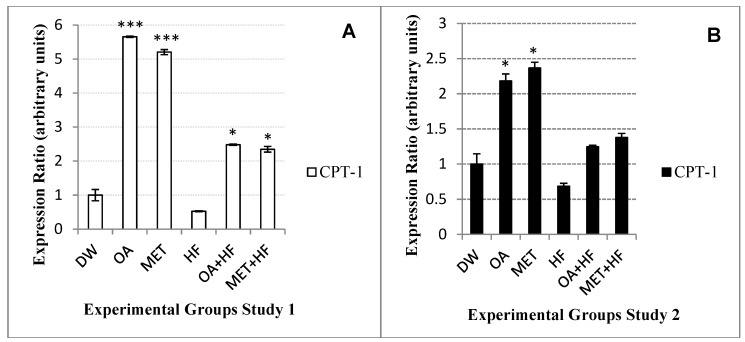
*Cpt-1* gene expression in the skeletal muscle of rats in Study 1 (**A**) and Study 2 (**B**) of the experiment. Each group was compared with DW (control group) (n = 3); *p* < 0.05(*) and *p* < 0.001 (***).

The OA dietary group increased the expression of *cpt-1* gene 5.6-fold higher than that of the control group, whereas the MET dietary group increased the expression to about 5-fold of the control group (Figure 7A). Both the OA + HF (*p* < 0.05) and MET + HF (*p* < 0.05) increased the expression of *cpt-1* to about 2-flod of the control group (Figure 7A). In both studies 1 and 2 of the experiment, the HF dietary group suppressed the expression of *cpt-1*to about a half of the control group (Figure 7A,B). In Study 1, the OA + HF and MET + HF groups had a 2-fold increased expression ratio of *cpt-1* compared to the control and a 4-fold increase compared to the HF group (Figure 7A).

In Study 2, the expression of *cpt-1* was increased over 2-fold in the OA (*p* < 0.05) and MET (*p* < 0.05) groups compared to the control group (Figure 7B). The OA + HF and MET + HF groups had a slightly increased expression of *cpt-1* as compared to the control group, but about a 2-fold increase compared to the HF group (Figure 7B). The MET and MET + HF groups had a higher expression of the *cpt-1* gene more than their comparable dietary groups OA and OA + HF (Figure 7B).

### 2.10. Effects Neonatal OA and MET Treatment on Expression of Glut-5

**Figure 8:**
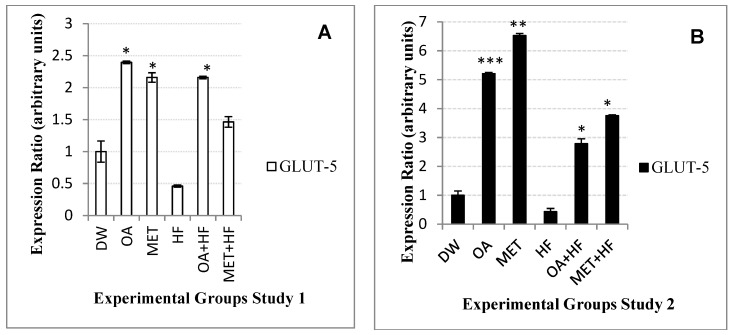
*Glut-5* gene expression in the skeletal muscle of rats in study 1 (**A**) and study 2 (**B**) of the experiment. DW was compared with each group (n = 3); *p* < 0.05 (*), *p* < 0.01 (**) and *p* < 0.001 (***).

In study 1, there was a 2-fold increase in the expression of *glut-5* in the rats that received neonatal OA (*p* < 0.05), MET (*p* < 0.05) and OA + HF compared to the control groups increased the expression of *glut-5* to 2-fold of the control group in Study 1 (*p* < 0.05; Figure 8A). The MET + HF group only increased the expression of *glut-5* just 0.4 ratio over the control group (*p* < 0.05; Figure 8A). In both studies 1 and 2 of the experiment, the HF dietary group decreased the expression of *glut-5* to half of the control group (Figure 8A,B). OA + HF group increased the expression of *glut-5* to 5-fold, whereas MET + HF increased it to 3-fold compared to the HF group (Figure 8A).

In the long term study of the experiment, the expression of *glut-5* gene was increased to 5-fold in the OA (*p* < 0.001) group and to 6-fold in the MET (*p* < 0.001) group compared to the control group (Figure 8B). The OA + HF (*p* < 0.05) increased the expression of *glut-5* to over 2.5-fold of the control, whereas the MET + HF (*p* < 0.01) group increased it to over 3.5-fold (Figure 8B). Furthermore, OA + HF increased *glut-5* expression to 6-fold of the HF group and MET + HF group increased it to 9-fold the ratio of HF group (Figure 8B).

### 2.11. Effects of Neonatal OA and MET Treatment on the Expression of Aldolase-b

**Figure 9:**
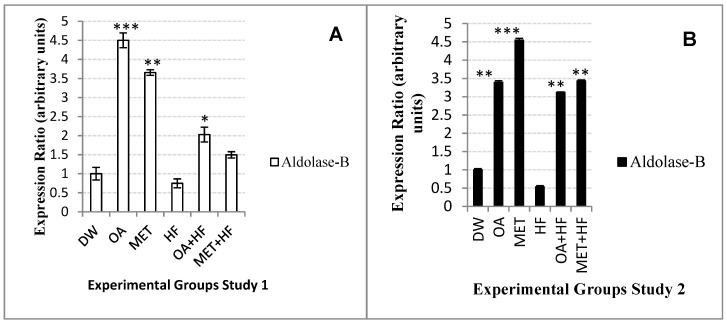
*Aldolase-b* gene expression in the skeletal muscle of rats in Study 1 (**A**) and Study 2 (**B**) of the experiment, derived from rats skeletal muscle samples. Each diet group was compared with the control group (DW) (n = 3); *p* < 0.05 (*), *p* < 0.01 (**) and *p* < 0.001 (***).

The OA group increased it to 4.5-fold (*p* < 0.001), MET group to 3.5-fold (*p* < 0.01), OA + HF group to 2-fold (*p* < 0.05) and MET + HF group to 1.5-fold of the control in the sub-acute study (Figure 9A). OA and OA + HF groups increased the expression of *aldolase-b* higher than their comparable dietary groups MET and MET + HF in study 1 (Figure 9A). In Study 1 of the experiment, the HF group decreased the expression of *aldolase-b* 2-fold lower than the OA + HF group and 1.5-fold lower than the MET+HF group (Figure 9A).

In the long term study, expression of *aldolase-b* was increased to about 3-fold in the OA (*p* < 0.01), OA + HF (*p* < 0.01) and MET + HF (*p* < 0.01) as compared to the control group, whereas MET + HF increased it to 4.5-fold of the control (Figure 9B). Moreover, in Study 2 *aldolase-b* expression was decreased 3-fold lower than the OA + HF in the HF and decreased over 3-fold in the MET + HF group (Figure 9B). However, in Study 2, MET (*p* < 0.001) and MET + HF (*p* < 0.01) increased *aldolase-b* expression more than OA and OA + HF did (Figure 9B) *Aldolase-b* gene expression was suppressed in the HF group only and increased in the other four dietary experimental groups compared to the control (Figure 9A,B).

## 3. Discussion

The energy expenditure of the body in the skeletal muscles requires glucose and lipid uptake, performs thermogenic functions and other biochemical processes in the body [14]. In the long term study of the experiment, the high fructose diet increased lipid storage in the triceps skeletal muscle tissue which had an increased fat deposition due to neonatal metabolic programming. Our findings showed that the rats that were treated with fructose in the neonatal period and later on in adulthood. The HF group gained more weight than rats from the other treatment groups at the end of the study. The excessive weight gain associated with high carbohydrate or fat diets impairs the ability of the body to increase fat oxidation [15]. Previous studies have shown that fructose causes the deposition of lipids into the skeletal muscles of both rats and humans [16,17].

Fructose consumption is known to induce metabolic effects associated with increased fat deposition [17] as we have confirmed with our experimental rats that were fed with HF, which eventually results in the development of obesity and metabolic syndrome. The rats in the HF group stored more fat as compared to most of the rats from the other groups. The metabolic effects of immoderate adiposity are exerted through the release of free fatty acids into peripheral and other tissues of the body [15].

In the sub-acute study, our findings showed metformin to be effective in reducing lipid storage in the MET + HF group. Similarly, in the long term (Study 2) experiments, metformin was shown to reduce lipid storage as seen in the group that received neonatal treatment with MET. Metformin treatment has also been found to reduce symptoms associated with obesity and metabolic syndrome in male mice [9].

Stearic acid was higher in the HF group, which is the group where obesity/metabolic dysfunctions were induced. These suggest that HF promotes accumulation of saturated free fatty acids in the body. As shown in the study, the elevated level of stearic acid could be responsible for the fructose-induced suppression of the *glut-4*, *nrf-1*, *cpt-1* and *ppary* genes. Saturated FFAs directly down-regulate the activity of *glut-4* and *ppary* genes [18]. Moreover, it was previously reported that a high- fructose diet causes suppression of the *glut-4* and *glut-2* gene expressions and this is dependent on both the diet and age at which the diet is administered [19,20]. However, in this study there were no differences in the suppression of *glut-4* between neonatal fructose treatments and adulthood fructose administration. We also showed that neonatal fructose consumption resulted in higher terminal body mass (Study 2 only) and saturated lipid accumulation within the skeletal muscle tissues (both studies 1 and 2 of the experiment). The development of long-term health risks such as obesity and diabetes are often a result of diets introduced to offspring during the critical window of developmental plasticity [21,22,23]. As such, administration of fructose in the neonatal period resulted in the development of health outcomes associated with metabolic syndrome in rats that further received dietary fructose as adults.

Stearic acid was the lowest in both OA + HF and MET + HF, suggesting that the OA and MET prevented the fructose induced accumulation of the saturated fatty acid in the body in both studies of the experiment. It has been reported that stearic acid and other long chain saturated FFAs in the high fat diet were responsible for suppression of *glut-4* gene [19]. Dodecanoic, myristic and palmitic saturated FFAs have also been shown to increase the risk factors of metabolic syndrome [24]. Polyunsaturated FFAs are important to the body and are acquired through dietary supplements as the body cannot synthesize them. Octadecadienoic acid, which is commonly known as lenoleic acid was a polyunsaturated free fatty acid detected in the samples by the GC-MS. Octadecadienoic acid is a long chain 18-carbon compound polyunsaturated FFA. The levels of oleic acid and octadecadienoic acid (unsaturated FFAs) were high in groups that received either OA and MET alone or a combination of both. In this study, supplementation with OA in the neonatal period promoted the accumulation of monounsaturated fatty acid in the body. Free fatty acids increase fat oxidation and glucose utilization [8]. Free fatty acids and glycerol are packaged in and transported by albumin from stored fats to be oxidized through major pathways such as the β-oxidation pathway, which also occurs in the mitochondria of skeletal muscles [25,26]. Monounsaturated FFAs; palmitoleic acid and oleic acid have also been found to have positive effects on health outcomes associated with metabolic syndrome [24,27].

Skeletal muscle tissue is a vital site for major glucose consumption, especially following a meal [23]. As such, glucose is transported into the tissue through an insulin stimulated facilitated diffusion by glut-4 [28,29,30,31]. In this study, the administration of both OA and MET in fructose fed rats increased the activity of *glut-4*, *cpt-1* and *nrf-1* genes. In order to achieve higher levels of *glut-4* expression within the skeletal muscles; transcription factors such as those belonging to the MEF2 domain, and *ppary* and *nrf-1* are required as *glut-4* gene expression is controlled at the transcriptional level [19]. *Ppary* controls the expression of *glut-4* transporter in skeletal muscle cells as it binds to MEF2C and activates it [32]. Higher expressions of the *nrf-1* gene increased the expression of *glut-4* gene, thereby increasing glucose transport capacity into the skeletal muscles [32]. As such, the high levels of both *nrf-1* and *glut-4* in our study confirms that glucose was being metabolized in the skeletal muscles from groups that had OA, MET, OA + HF and MET + HF in both studies of the experiment.

The gene expression of both *fas* and *acc-1* were suppressed in the OA, MET, OA + HF and MET + HF in both the phase 1 and 2 experiments. However, in the HF group, the expressions of *fas* and *acc-1* were increased. When animals are well fed on carbohydrates, fatty acid oxidation is lowered by regulatory hormones such as insulin, but fatty acid synthesis is increased [25]. This suggests that the HF dietary groups promoted FFA synthesis in the skeletal muscle cells. Phosphorylation of *acc-1* by AMPK decreases *acc* activity and malonyl-CoA content in rats [33,34,35]. Studies have shown that fatty acid synthesis is also regulated through long-term hormonal regulation and or short-term substrate availability where the presence of polyunsaturated FFAs in the diet decreases the concentration of *acc-1* and *fas* [18]. Moreover, in this study, the presence of polyunsaturated FFAs in the OA, MET, OA + HF and MET + HF groups correlated with the decreased expression levels of *acc-1* and *fas*.

Findings from the current study showed that *cpt-1* gene was highly expressed in both OA and MET groups and was suppressed in the HF group. *Cpt-1* catalyzes the rate limiting step in skeletal muscle fatty acid oxidation, in which decreased cytosolic long chain fatty acid Co-A results in the increase of the activity of *acc-1*, where malonyl Co-A is phosphorylated and decreases the activity of *cpt-1* in which long acid chain fatty acid carnitine is ceased to be produced and will not be transferred to the mitochondria for oxidation [7].

*Glut-5* isoform gene is another hexose transporter found in the body. Although *glut-5* is not highly abundant in the skeletal muscle tissues, it has high affinity for fructose and low affinity for glucose, therefore it is important for both fructose and glucose homeostasis. After consumption of high fructose diet, fructose is efficiently metabolized by ketohexokinase into fructose-1 phosphate and then cleaved by *aldolase-b* into dihydroxyacetone phosphate and glyceraldehyde [16,17,36]. Dihydroxyacetone phosphate can directly enter gluconeogenesis; whereas glyceraldehyde is phosphorylated into glyceraldehyde-3 phosphate [17,36]. Glyceraldehyde-3 phosphate can either be used for glycolysis, gluconeogenesis, or enter the pentose phosphate pathway and be metabolized into acetyl-CoA so that it can be used for liponeogenesis [17,36].

There was a decreased expression of *glut-5* in the HF group and this was associated with high levels of saturated FFAs. The *glut-5* gene expression was also high in the OA, MET, OA + HF and MET + HF treated groups. Over expression *glut-5* in non-insulin dependent diabetes is associated with increased fructose absorption [23,36]. In this study, over expression of *glut-5* could have also been influenced by the rate at which fructose was metabolized. This correlated to the increased expression of *aldolase-b* in OA, MET, OA + HF and MET + HF groups when compared with normal control and HF groups. However, the suppression of both glut-5 and *aldolase-b* in the HF suggests that HF could lead to fructose malabsorption.

## 4. Materials and Methods

### 4.1. Ethical Clearance

The experiments were conducted on 96 female Sprague Dawley pups that were housed in the Central Animal Services at the University of the Witwatersrand. Ethical clearance for animal use was granted by the Animal Ethics Committee of the University of the Witwatersrand (Ethics Screening Number 2014/47/D).

### 4.2. Experimental Design

The study was conducted in two main experimental studies, a sub-acute study and a long term study. Study 1 of the experiment was between postnatal days 7–14. The seven-day old pups were randomly assigned to the following treatment groups: control—distilled water (DW) and 0.5% (*v*/*v*) dimethyl sulphoxide, oleanolic acid (60 mg/kg) (purity > 99%) [37] dissolved in 0.5% (*v*/*v*) dimethyl sulphoxide, metformin (500 mg/kg) (purity > 98%) [38] 0.5% (*v*/*v*) dimethyl sulphoxide, high fructose solution (HF; 20% (*w*/*v*)—dissolved in 0.5% (*v*/*v*) dimethyl sulphoxide, oleanolic acid (OA) + high fructose diet (OA + HF), and metformin + high fructose diet (MET + HF). Treatments were administered once daily via oral gavage (10 mL/kg body weight) between 09:00–10:00 a.m. The pups were allowed to continue nursing with their dams freely during the experimental period. The dosages of oleanolic acid, metformin and high fructose died were based on the less toxic levels obtained from previous studies using these doses. On PD 14, the rat pups were euthanized and samples collected. The dams were returned to stock. In the second study of the experiment, the rats were weaned onto normal rat chow and plain drinking water on PD21. The pups were then housed individually from PD 22 in cages where they had *ad libitum* access to rat chow and plain drinking tap water until PD112.

### 4.3. Terminal Procedure and Sample Collection

On PD 112, the rats were then euthanized by an intra-peritoneal injection of sodium pentobarbital (200 mg/kg body mass; Eutha-naze^®^ (Bayer Corporation, Johannesburg, South Africa). The triceps skeletal muscles were dissected out, placed in cryovials, then snap frozen in liquid nitrogen and stored at −80 °C freezer until analyses.

### 4.4. Lipid Extraction, Purification and Identification by Gas Chromatography-Mass Spectrometry

Lipid extraction and purification from the skeletal muscle samples from study 1 and 2 was performed according to a modified method described by [39]. Briefly, the snap frozen tissue samples were taken from the −80 °C and placed on ice. Thereafter, the muscle tissue was cut from each sample with a clean scalpel blade and 50 mg was weighed. Microcentrifuge tubes were used to place the 50 mg muscle tissues. An internal standard (IS) mixture (Table 5) was prepared and added to the weighed muscle tissue. The IS consists of methanol, water and chloroform. Steel beads were then added to the sample mixture and the samples were placed on a Retch M400 vibration mill for homogenization. This was followed by the removal of steel beads from the sample, where the sample mixture was transferred into a new clean tube. The after homogenization, the internal standard mixture (IS) was added into the sample (Table 5), then vortexed for 60 s using the Vortex-2 Genie machine (Vortex-Genie^®^ 2, Scientific Industries, Inc. Bohemia, New York, NY, USA). After that the sample mixture was then centrifuged for 10 min at 2000× *g* at 4 °C. This resulted in a phase separation, with the clear polar layer at the bottom and the milky apolar layer at the top of the muscle pellet. Both polar and apolar samples were used for analysis, where in each sample 725 µL of polar sample was used and 400 µL of apolar sample was used and transferred into Agilent vials (Table 5). Each sample had five replicates. The samples were then dried under nitrogen. An oximation solution was then prepared by dissolving 200 mg of methoxyamine into 10 mL of pyridine. In each dried sample, 50 µL of the oximation solution was added followed by a 60 s vortexing. The samples were then placed at room temperature for an hour. This was followed by the addition of 50 µL of bistri fluoacetamide (BSTFA), which contained 1% of trimethylsilyl chloride (TMCS) and then incubated for one hour at 40 °C. An external standard was then prepared using 50 µL of 30 µg/mL eicosane in hexane, which was added into the agilent vials inserts of each sample before running the GC-MS analysis.

The response factor of each lipid analysed was calculated using:

(1) $$Ra=\frac{Aa}{ma}$$

where *ma* is the mass of the lipid analyte of the free fatty acid *a* and *Aa* is the corresponding peak area of the free fatty acid a and *Ra* is the Response Factor or the Peak Area Ratio of the free fatty acid *a*.

### 4.5. RNA Extraction

Samples were randomly selected from rats in Study 1 and Study 2 experimental groups, and a total of 12 samples were used per extraction. From each sample, muscle tissue was crushed in a mortar with a pestle using liquid nitrogen and 200 mg was weighed on a scale. The weighed tissue was then transferred into a 1.5 mL tube and dissolved in a 1 mL Trizol (TRIzol^TM^ Reagent, Thermo Fisher Scientific Inc., Waltham, MA, USA). This was followed by homogenization and incubation on ice for 5 min, and then centrifugation at 12,000× *g* at 4 °C for 15 min. Trizol was then discarded from the sample homogenate. Chloroform (200 µL) was added into the sample homogenate using a pipette, then incubated on ice for 15 min. Thereafter, the sample homogenate was centrifuged at 12,000× *g* for 15 min in order to achieve phase separation. The aqueous phase was then transferred into a 2 mL tube. Isopropanol (0.5 mL) was used to precipitate the RNA, followed by incubation on ice for 10 min. The sample was then centrifuged for 10 min at 12,000× *g* at 4 °C. After centrifugation, the supernatant was removed from the sample and the RNA sample pellet was air dried. RNase free water (20 µL) was then used to dissolve the extracted RNA pellet. This was followed by determination of RNA concentration and ratio using a nanodrop machine (NanoDropTM Lite Spectrophotometer, Thermo Fisher Scientific Inc.).

### 4.6. Gel Electrophoresis

**Figure 10:**
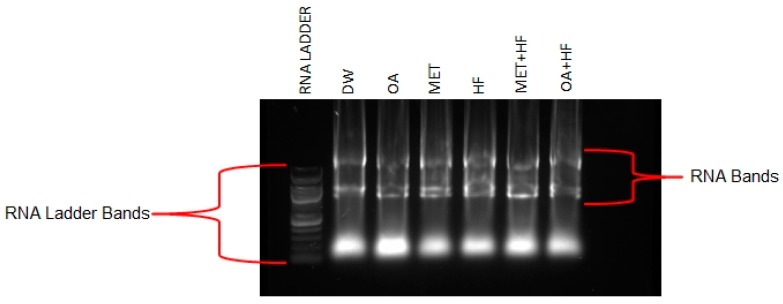
The RNA bands of the 6 experimental groups. RNA ladder 50 kb.

Gel preparation involved weighing 1.5 g of agarose gel on a scale and dissolving it in 150 mL of tris acetate buffer (1× TAE buffer), which was prepared at 500 mL TAE buffer. The solution was then microwaved for 60 s to dissolve the gel. The gel solution was cooled at room temperature to 50 °C temperature. Ethidium bromide (2 µL) was then added to the gel solution. The gel was poured into the electrophoresis chamber, in which sample wells were created using the comp. One extracted RNA sample from each experimental group (DW, OA, MET, HF, OA + HF and MET + HF) was randomly selected to be used for the gel electrophoresis, in which a total of six samples were used. Two were from Study 1 and three from Study 2 (DW and MET = study 1 and OA, HF, OA + HF and MET + HF = study 2). A staining procedure then followed, were in each sample a pipette was used to obtain 1 µL of RNA, which was mixed with a 1 µL of purple dye. RNA ladder standard marker was also used at a 1:1 ratio with the purple dye. The RNA marker was added into the first sample well, followed by DW, OA, MET, HF, OA + HF and MET + HF in that order. The rest of the TAE buffer solution was added into the electrophoresis chamber as running buffer. The gel was then run on an electrophoresis chamber for 80 min at 60 volts. RNA bands were then visualized at the SYBR Green Filter at 500–600 m (Figure 10).

### 4.7. cDNA Synthesis

DNA was synthesized from RNA samples using the Superscript VILO cDNA synthesis kit (iScriptTM select cDNA synthesis kit, Bio-Rad Laboratories, Inc., Foster city, CA, USA). This involved determining the RNA concentration needed for synthesizing cDNA, which varied per sample as it depended on the RNA concentration observed from the Nanodrop machine. A master-mix for each single reaction was prepared using 4 µL of 5× VILO reaction mix, 2 µL 10× Superscript enzyme mix, sample RNA of up to 2.5 µg and DEPC-treated water of up to 20 µL. The mixture was then incubated at 25 °C for 10 min. Incubation was repeated at 42 °C for an hour. The reaction was terminated at 85 °C incubation for 5 min.

### 4.8. Quantitative Real Time PCR (RT-qPCR)

Quantitative Real Time PCR (qPCR) was performed for seven genes, namely; *acc-1*, *cpt-1*, *fas*, *nrf-1*, *glut-4*, *glut-5* and *aldolase-b*. For each gene, three replicates per sample from each experimental group were analyzed. Firstly, 1 µL of sample cDNA was pipetted from the sample and diluted with 19 µL of RNase free water to get a 20 µL volume. From the diluted cDNA sample, 1:20 ratio of cDNA to RNase free water was pipetted into new three qPCR tubes. The gene of interest forward and reverse primers and those of reference gene master-mix were prepared separately, with SYBR green master-mix. Primers used are provided in Table 6.

The primer stocks were diluted according to the manufacturer’s guide. From the master-mix of each gene, 7 µL was pipetted into the qPCR sample tubes. Thereafter, cDNA (3 µL) was added into the qPCR sample tube in order to achieve a 10 µL final volume. The samples were loaded into the RT-qPCR machine for analysis in a specific order of choice. The RT-qPCR was set to run using instructions provided by the manufacturer as the instrument user guide, in which a standard cycling mode was used.

### 4.9. Statistical Analysis

Data was expressed as mean ± standard deviation (SD). A one way ANOVA was used to determine differences in means from different experimental groups. All statistical analyses were performed in R version 3.4.0 statistical software (R Development Core Team, Auckland, New Zealand) Level of significance was set at *p* ≤ 0.05.

## 5. Conclusions

The present study showed that neonatal intake of oleanolic acid and metformin almost equally prevented the fructose induced metabolic dysfunction in the rats, sub-acutely and in the long term. Though metformin is an already known drug that is used in the treatment of type 2 diabetes across the globe, this study showed its ability to potentially prevent metabolic disorders when administered during a period of developmental plasticity. In a similar vein, the study also showed the efficacy of a plant-derived compound, oleanolic acid as prophylactic agent in the prevention of metabolic dysfunction caused by high dietary fructose intake. These results show that there is need to further develop strategies to identify candidate biologically active phytochemicals for use in critical developmental windows for prophylactic interventions to help to reduce the occurrence of metabolic diseases such as type2 diabetes and obesity.

## Figures and Tables

**Table 1:** Study 1 weight of the rats during the neonatal developmental stage (day 1 to day 14), presented in mean (g) ± SD.

Experimental Groups	Induction Body Mass (g)	Terminal Body Mass (g)
DW	15.11 ± 1.54	17.01 ± 1.71
OA	16.60 ± 1.22	18.60 ± 0.86
MET	15.79 ± 1.42	16.75 ± 1.85
HF	16.46 ± 1.42	18.15 ± 1.31
OA + HF	16.14 ± 2.26	17.78 ± 2.52
MET + HF	16.24 ± 1.96	17.01 ± 1.71

**Table 2:** The response factor (*Ra*) of stearic acid in both sub-acute (study 1) and long term (study 2) of the experiment.

Experimental Groups	Study 1 (Mean ± SD)	Study 2 (Mean ± SD)
DW	2.30 ± 1.72	1.29 ± 0.77
OA	1.01 ± 1.04	0.75 ± 0.89 *
MET	0.75 ± 0.89 *	0.38 ± 0.23 **
HF	4.70 ± 7.19 *	1.92 ± 1.31 *
OA + HF	0.56 ± 0.48 **	0.37 ± 0.17 **
MET + HF	0.24 ± 0.19 ***	0.20 ± 0.16 ***

Data of the stearic acid response factor (*Ra*) were calculated in R statistics and presented as mean ± sd (n = 5). Student’s *t*-test for phase 1, MET versus DW (control) *p* < 0.05 (*), HF versus DW *p* < 0.05 (*), OA + HF versus DW *p* < 0.01 (**) and MET + HF versus DW *p* < 0.001 (***).In phase 2, OA versus DW *p* < 0.05 (*), MET versus DW *p* < 0.01 (**), HF versus DW *p* < 0.05 (*), OA + HF versus DW *p* < 0.01 (**) and MET + HF versus DW *p* < 0.001 (***).

**Table 3:** The response factor of oleic acid in the rats from the sub-acute and long term experiments.

Experimental Groups	Study 1 (Mean (*Ra*) ± SD)	Study 2 (Mean (*Ra*) ± SD)
DW	0.00 ± 0.00	0.00 ± 0.00
OA	1.55 ± 1.03^***^	1.39 ± 0.26^***^
MET	0.00 ± 0.00	0.67 ± 0.47^***^
HF	0.00 ± 0.00	0.00 ± 0.00
OA + HF	0.09 ± 0.08^*^	0.17 ± 0.03^*^
MET + HF	0.00 ± 0.00	0.06 ± 0.03

Data of the oleic acid response factor (*Ra*) were calculated in R statistics and presented as mean ± sd (n = 5). In phase 1, student’s *t*-test results for OA versus DW *p* < 0.001 (***) and OA + HF versus DW *p* < 0.05 (*). In phase 2, OA versus DW *p* < 0.001 (***), MET versus DW *p* < 0.001 (***) and OA + HF versus DW *p* < 0.05 (*).

**Table 4:** Octadecadienoic acid response factor (*Ra*) from rats in studies 1 and 2 of the experiment.

Experimental Groups	Study 1 (Mean (*Ra*) ± SD)	Study 2 (Mean (*Ra*) ± SD)
DW	0.35 ± 0.33	0.43 ± 0.58
OA	2.96 ± 3.12 ***	1.78 ± 1.06 ***
MET	1.07 ± 0.60 ***	0.75 ± 0.58
HF	0.08 ± 0.09 *	0.00 ± 0.00
OA + HF	0.43 ± 0.58	0.24 ± 0.17
MET + HF	0.51 ± 0.53	0.20 ± 0.15

Data of the octadecadienoic acid response factor (*Ra*) were calculated in R statistics and presented as mean ± sd (n = 5). In phase 1, student’s *t*-test *p*-values were as follows; OA versus DW *p* < 0.001 (***), MET versus DW *p* < 0.001 (***) and HFruD versus DW *p* < 0.05 (*). In phase 2, OA versus DW *p* < 0.001 (***).

**Table 5:** Standard Internal Mixture.

Internal Standard Mix (IS)			
Weight (mg)	Methanol (µL)	Water (µL)	IS (µL)
50	400	75	50
**After Homogenization**			
Water (µL)	Chloroform (µL)	Total polar (~µL)	Total apolar (~µL)
200	400	725	400

**Table 6:** Specific primers used for each gene.

Gene For:	Primers Orientation	
	Forward 5′–3′	Reverse 3′–5′
*cpt-1*	GCA AAC TGG ACC GAG AAG AG	TCC ATG AGG GAT GGA CTC TC
*nrf-1*	GTT GGA TCC CTC TCA CCC ATT G	CCA AGT CGA GAC TTA ATT CC
*acc-1*	AGG AGG GAA GGG AAT CAG AA	TGT GCT GCA GGA AGA TTG AC
*Fas*	GCT TTG CTG CCG TGT CCT TCT	GTG TCT GCT GGG GTC CTC GTT
*glut-4*	GCA GCG AGT GAC TGG AAC A	CCA GCC ACG TTG CAT TGT AG
*glut-5*	CCA GAG AAG CAT GGA GCA	AGG ATG ACC CAA AGG CAG
*Aldolase-b*	GCC ACC TCA CAC AGC TTC TG	TCG GTG AGC CAT GAT GAC A
*Actin*	AGC CAT GTA CGT AGC CAT CC	CTC TCA GCT GTG GTGGTG AA
*Gapdh*	GCA CAG TCA AGG CCG AGA AT	GCC TTC TCC ATG GTG GTG AA
